# Time From Referral to Discharge From High Secure Care: Challenges for Flow Through the Forensic Estate

**DOI:** 10.1192/bjo.2024.260

**Published:** 2024-08-01

**Authors:** Gavin Third, Cheryl Rees, Lindsay Thomson, Lindsay Gilling McIntosh

**Affiliations:** 1 The State Hospital, Carstairs, United Kingdom; 2 University of Edinburgh, Edinburgh, United Kingdom

## Abstract

**Aims:**

The Independent Forensic Mental Health Review (Scottish Government, 2021) highlighted an issue with timely transitions through and out of Scottish forensic inpatient services. Concerns were raised regarding the impact of transfer and discharge delays upon patients. As part of a wider service evaluation examining the pathways forensic mental health patients navigated through secure inpatient care, this study aimed to identify the requirements, processes and time-frames involved in transfer from The State Hospital (TSH), which provides male only, high secure care to Scotland and Northern Ireland.

**Methods:**

Data for 69 patients noted on TSH transfer list (2017–2019) were collected. In addition to patient demographic, clinical and forensic variables, data was gathered about use of appeals against excessive security under section 264 and 265 of the Mental Health (Care and Treatment) (Scotland) Act 2003.

**Results:**

Forty-nine (71.0%) patients were referred to medium secure care, 6 (12.2%) to low secure care and 14 (20.3%) for return to prison. Schizophrenia was the most common primary diagnosis (43, 62.3%), with 75.5% (37) of those referred to medium secure care vs 21.4% (3) returning to prison having received this diagnosis. There were statistically significant associations in terms of time between referral and transfer between individuals who had a primary diagnosis of Schizophrenia/Schizoaffective disorder (no 114, yes 388.5 days; Median) and whether they had lodged a section 264 appeal (no 109.5, yes 469.0 days; Median) or section 265 appeal (no 134.5, yes 517.0 days; Median) against excessive security. There were no significant differences in days from referral to transfer/discharge based on behaviour leading to admission or the number of formal attempts to transfer during current admission. Twenty (40.8%) patients referred to medium secure services made a successful section 265 appeal which resulted in a ruling that they should be transferred within three months. Seven (35%) of these patients were transferred inside three months.

**Conclusion:**

Patients are waiting significantly variable lengths of time from referral to transfer depending on the service they are being referred to. The use of section 264 and 265 appeals against excessive security was implicated in a greatly increased length of time to transfer. Patients considered to have the most serious chronic mental health conditions are waiting the longest time for transfer with potential implications for their mental health. Patients’ human rights are potentially affected due to continuing to be placed in conditions of excessive security for more than a year following decision to refer.

